# Interleukin-6 in Cardiogenic Shock

**DOI:** 10.1007/s11886-025-02273-0

**Published:** 2025-08-30

**Authors:** Luca Fazzini, Michele Golino, Francesco Moroni, Matteo Castrichini, Gregorio Tersalvi, Paolo Manca, Antonio Abbate

**Affiliations:** 1 Department of Cardiovascular Diseases, Mayo Clinic, Rochester, MN United States of America; 2 Department of Medical Sciences and Public Health, Clinical Cardiology Unit, University of Cagliari, Cagliari, Italy; 3 Pauley Heart Center, Virginia Commonwealth University, Richmond, VA United States of America; 4 Robert M. Berne Cardiovascular Research Center, University of Virginia, Charlottesville, VA United States of America; 5 Department of Internal Medicine, University of Virginia, Charlottesville, VA United States of America; 6 Department of Medicine, Università Milano-Bicocca, Milan, Italy; 7 Department of Clinical Cardiology and Heart Failure, Istituto Mediterraneo per i Trapianti e Terapie ad Alta Specializzazione, ISMETT IRCCS, Palermo, Italy

**Keywords:** Myocardial infarction, Heart failure, Inflammation, Cytokines, Interleukin-6

## Abstract

**Purpose of Review:**

Cardiogenic shock (CS) is one of the leading causes of mortality in patients with acute cardiac disease, and systemic inflammation plays a critical role in its pathophysiology. This review explores the role of interleukin-6 (IL-6) in CS, with a focus on its biological pathways, prognostic value, and potential as a therapeutic target, highlighting the importance of addressing inflammation in this context.

**Recent Findings:**

Recent evidence highlights systemic inflammation implied in CS progression. Among the various cytokines involved, IL-6 is a major pro-inflammatory cytokine associated with organ dysfunction, and high mortality rates in acute myocardial infarction complicated by CS. Mechanistic studies exhibited an involvement of IL-6 since the first stages of CS onset, suggesting its role as both a biomarker and likely a mediator of CS.

**Summary:**

IL-6 emerges as a key inflammatory mediator in CS pathophysiology, serving as a prognostic biomarker and a potential therapeutic target. Future research should focus on further understanding the underlying mechanism linking acute inflammation and CS, patient phenotyping, and optimizing anti-inflammatory strategies investigating IL-6-targeted therapies to improve outcomes in this poor prognosis condition. Clinical trials of IL-6 blockade in cardiogenic shock are lacking.

## Introduction

Cardiogenic shock (CS) is characterized by insufficient cardiac output due to severe cardiac dysfunction, leading to tissue hypoperfusion, venous congestion, and multiorgan dysfunction [1]. Despite advancements in therapeutic strategies, including timely reperfusion and the implementation of early mechanical circulatory support, it complicates 5–10% of acute myocardial infarction cases (AMI-CS) and remains the leading cause of post-AMI mortality (> 40%) [1–5].

While AMI-CS has historically represented the most studied form of CS, heart failure-related CS is increasingly recognized as a significant contributor to the modern CS landscape [6]. Indeed, the current evidence remains largely derived from studies focused on AMI-CS.

Hypoperfusion in the setting of CS, combined with tissue damage and metabolic imbalances, causes progressive organ dysfunction [1, 7] and triggers systemic inflammation through damage-associated molecular patterns [8]. Cytokines like interleukin-1 (IL-1), IL-6, and tumor necrosis factor-alpha (TNF-α), are the main drivers of CS-associated systemic inflammation and lead to its progression [9–11]. IL-6, in particular, has emerged as a central mediator in the systemic response of CS, further contributing to myocardial injury progression, cardiac dysfunction, vasodilation, and multiorgan failure. Targeting the IL-6 pathway could offer a promising defensive strategy in CS. This review explores the role of IL-6 in the systemic inflammation associated with CS and the potential therapeutic implications of targeting its pathway.

## Cardiogenic Shock, Vasodilation, and Systemic Inflammation

**Fig. 1:**
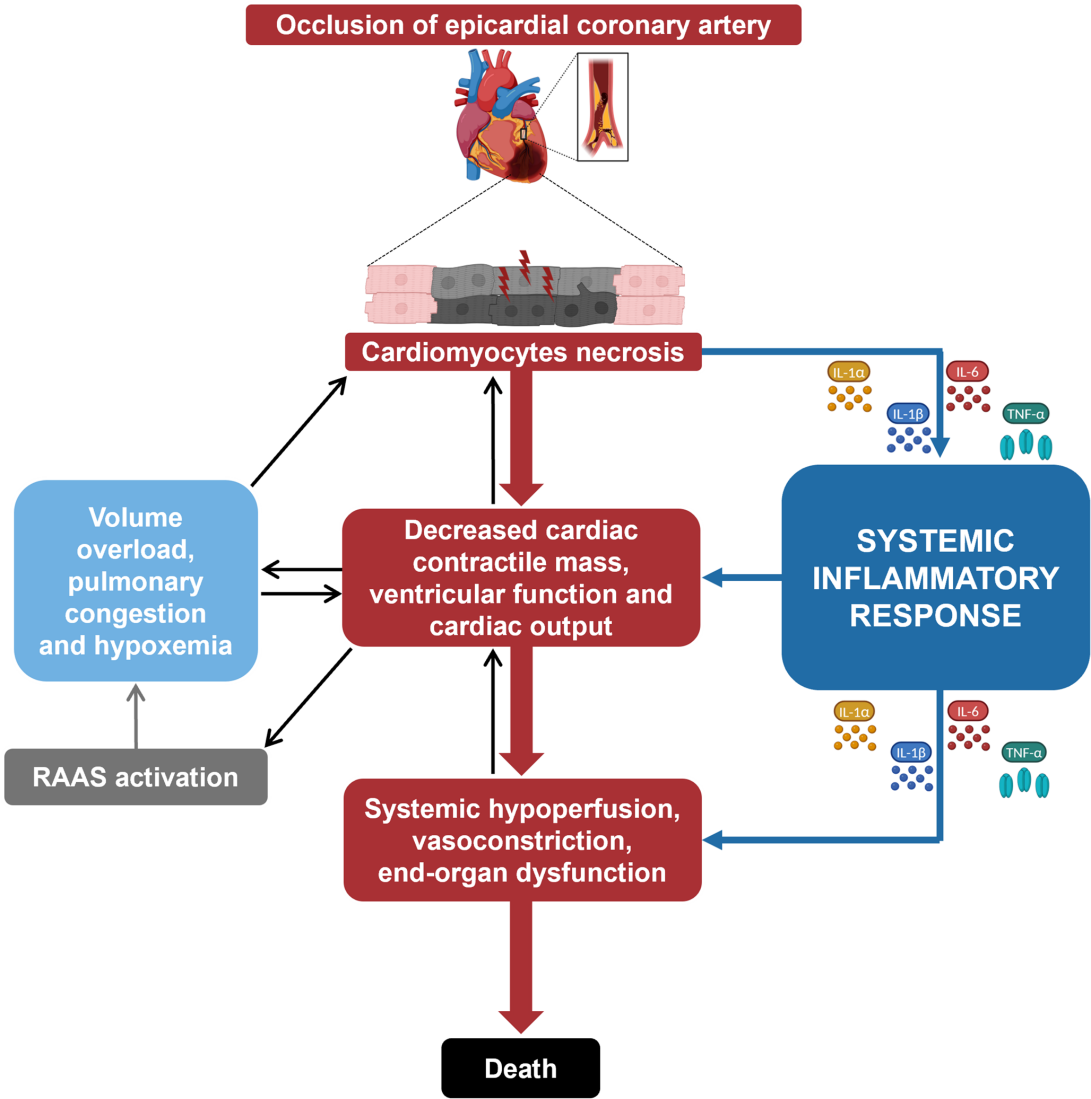
Progression from acute myocardial infarction to cardiogenic shock and the role of systemic inflammatory response. During acute myocardial infarction (AMI), the occlusion of the epicardial coronary artery leads to cardiomyocyte necrosis. The necrosis is associated with a release of damage-associated molecular patterns, directly contributing to the activation of the innate immune system. Necrosis and systemic inflammation cause a decrease in cardiac contractile mass, ventricular function, and cardiac output, which lead to systemic hypoperfusion, vasoconstriction, and end-organ dysfunction. The latter further decreases cardiac output, creating a negative feedback loop. Additionally, decreased cardiac output results in volume overload, pulmonary congestion, and hypoxemia, further impacting cardiac function and activating the renin-angiotensin-aldosterone system (RAAS). The RAAS activation, triggered by reduced renal perfusion, causes vasoconstriction, sodium and water retention, and aldosterone release, exacerbating fluid overload. This cascade results in a systemic inflammatory response characterized by the release of inflammatory cytokines such as interleukin (IL)-1α, IL-1β, IL-6, and tumor necrosis factor (TNF)-α. These three cytokines contribute to further systemic inflammation and organ dysfunction, perpetuating the cycle of cardiac and systemic deterioration during AMI and cardiogenic shock, potentially leading to death. They also stimulate the production of angiotensinogen in the liver, upregulate renin release, and angiotensin II production. Angiotensin II, in turn, promotes more cytokine release, creating a vicious cycle. (Created in BioRender. Golino, M. (2025) https://BioRender.com/ao97t7j)

Patients with AMI-CS often develop a systemic inflammatory response (SIRS) associated with vasodilation independent of concurrent infection, mimicking a sepsis-like phenotype [1, 12, 13]. Systemic inflammation is common in AMI-CS, triggered by either organ hypoperfusion, metabolic impairment, or due to infection [12, 14]. Concomitant sepsis occurs in 10–20% of AMI-CS cases, further worsening outcomes [13, 15]. Nonetheless, it has been shown that the low systemic vascular resistance might persist despite the administration of vasopressors, with this condition being noted at the onset of shock several days before sepsis was suspected, suggesting a systemic inflammation triggered by AMI itself as a potential cause [13]. Notably, patients with SIRS and CS have worse outcomes [16, 17]. Additionally, many patients with CS still experience high mortality rates even after normalization of the cardiac index and without any evident infection. This seems to be due to the ongoing activation of systemic inflammatory pathways, sustaining the spiral of shock [13, 18]. Upon coronary vessel occlusion, the cardiomyocytes supplied by that vessel incur necrosis. If the area of necrosis is sufficiently large to impair overall cardiac contractility, cardiac output is reduced, leading to systemic hypoperfusion, pulmonary congestion, hypoxia, and eventually end-organ failure. In this setting of AMI-CS, SIRS is triggered by necrosis markers, metabolic impairment, and becomes self-perpetuating, driven by ongoing systemic hypoperfusion and multiorgan dysfunction. (Fig. 1).

Cytokines are small signaling proteins primarily secreted by activated immune cells that act as key mediators in intercellular communication and play a central role in regulating inflammation and immune responses. They are biochemical mediators and regulate immune responses via pro-inflammatory and anti-inflammatory pathways, potentially serving as therapeutic targets. Cytokines exert their effects through interaction with specific receptors on target cells, which initiate downstream signaling cascades that modulate immune cell proliferation, differentiation, and function [19, 20]. Over the last twenty years, cytokines, especially IL-6, have been studied in the context of CS. However, the exploration of the interleukin family in CS is still limited, with previous investigations mainly focusing on AMI, atherosclerosis, heart failure, and inflammatory cardiomyopathies [21].

## IL-6 Biology

IL-6 is a pleiotropic cytokine primarily secreted by monocytes, macrophages, endothelial cells, fibroblasts, and vascular smooth muscle cells [22]. It primarily acts in the systemic inflammatory acute response, stimulating the liver to produce C-reactive protein (CRP), mannose-binding lectin, and complement components [22–24]. CRP is an acute-phase protein synthesized by hepatocytes in response to pro-inflammatory cytokines, particularly IL-6, which promotes damaged host cell clearance through opsonization and complement activation. Similarly, mannose-binding lectin and complement components are proteins of the innate immune system that primarily recognize pathogens, promoting their elimination through opsonization and activation of the complement cascade [25].

**Fig. 2:**
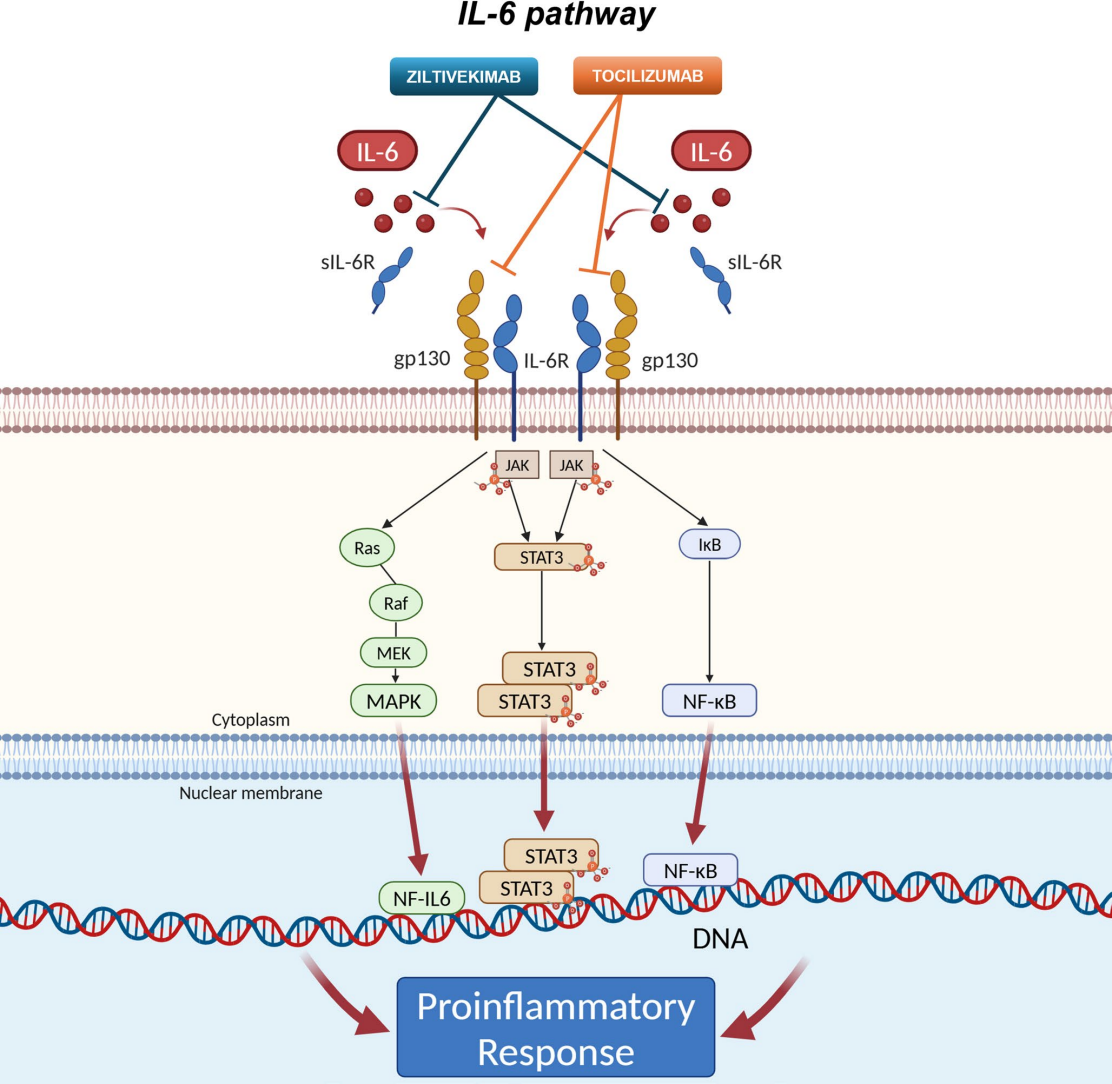
Interleukin-6 pathway and the drugs targeting its modulation. Interleukin (IL)-6 plays a central role in the inflammatory response associated with cardiogenic shock. IL-6 binds to its membrane-bound IL-6 receptor (IL-6R), and this complex associates with gp130, initiating intracellular signaling (classical signaling). In trans-signaling, IL-6 binds to soluble IL-6R (sIL-6R), and this complex interacts with gp130 on cells that do not express membrane-bound IL-6R. The IL-6/IL-6R/gp130 complex activates Janus kinases (JAKs), which phosphorylate and activate the signal transducer and activator of transcription 3 (STAT3). Phosphorylated STAT3 dimerizes and translocates to the nucleus to induce pro-inflammatory gene transcription. The complex can also activate the Ras/Raf/Mitogen-Activated Protein Kinase (MAPK) cascade, activating transcription factors like Activator Protein 1 (AP-1). Additionally, activation of gp130 can lead to Inhibitor of nuclear factor kappa B (IκB) degradation, releasing NF-κB to enter the nucleus and promote pro-inflammatory gene expression. Tocilizumab, a monoclonal antibody, blocks IL-6R, while ziltivekimab, also a monoclonal antibody, targets and inhibits IL-6 ligand. (Created in BioRender. Golino, M. (2025) https://BioRender.com/t51d676)

IL-6 signaling occurs via a classical pathway, mediated by membrane-bound IL-6 receptors, and a trans-signaling pathway, where IL-6 binds to soluble receptors, activating the cells that lack IL-6 receptors [23]. During the acute phase of CS, systemic hypoperfusion and metabolic derangements lead to endothelial dysfunction, blood vessel leakage, and the activation of immune cells, including granulocytes and monocytes, which contribute to IL-6 overproduction. IL-6 activates intracellular signaling pathways via complex trans-signaling pathways and its receptor complex composed of IL-6R and glycoproteins, ultimately driving pro-inflammatory gene transcription through Signal Transducer and Activator of Transcription 3 (STAT3), Mitogen-Activated Protein Kinase (MAPK), and Nuclear Factor Kappa-Light-Chain-Enhancer of Activated B Cells (NF-κB) pathways. As a result, regulation of the tissue damage, induction of adhesion molecules, further recruitment of inflammatory cells, and inhibition of T-cell apoptosis and regulatory T-cell differentiation contribute to the amplification and persistence of the acute inflammatory response [26]. Figure 2 illustrates IL-6 pathways, signaling, and effects.

## IL-6 and Acute Myocardial Infarction-Related Cardiogenic Shock (AMI-CS)

### IL-6 and Clinical Findings

Few studies have investigated IL-6 levels during AMI-CS during the last twenty years. In a prospective comparative study enrolling patients between 2001 and 2003, IL-6 levels were assessed in patients admitted with ST-segment elevation myocardial infarction (STEMI) with or without AMI-CS. Those who developed AMI-CS (*N* = 19, 46%) exhibited significantly elevated levels of IL-6 compared to patients with uncomplicated AMI. A SIRS was defined by the coexistence of at least two of the following: fever (body temperature > 38 °C)/hypothermia (< 36 °C), heart rate > 90 beats/min, respiratory rate > 20 breath/min or arterial partial pressure of carbon dioxide (PaCO2) < 32 mmHg, leukocytosis (> 12,000 cells/mm3)/leukopenia (< 4,000 cells/mm3). Notably, the patients with both AMI-CS and SIRS (*N* = 7) exhibited the highest levels of IL-6 [11]. Specifically, uncomplicated MI exhibited IL-6 peak levels of 34 pg/mL, significantly lower when compared to AMI-CS and AMI-CS with SIRS (109 pg/ml, *p* = 0.003, 253 pg/ml, *p* < 0.001, respectively). In another study, *Selejan et al.* demonstrated higher expression of IL-6 and TNF-α in monocytes through intracellular cytokine staining in patients with AMI-CS compared to uncomplicated AMI or age-matched healthy controls [27]. Similarly, white blood cell count and cytokine levels, including IL-1β, IL-5, IL-6, IL-10, TNFα, interferon (IFN)γ, Monocyte Chemoattractant Protein (MCP)-1, and eotaxin (CCL11) were prospectively and longitudinally assessed throughout the course of CS (50% were AMI-CS) to characterize the acute inflammatory response in this context. IL-6 levels, compared to controls, were already elevated on the first day and demonstrated a positive correlation with NT-proBNP levels (r^2^ = 0.346, *p* = 0.004) and norepinephrine use (r^2^ = 0.20, *p* = 0.03), while showing a negative correlation with systemic mean blood pressure (r^2^ = 0.293, *p* = 0.006) [12]. These studies support the potential role of IL-6 in CS progression and hemodynamic deterioration. The IL-6, along with CRP and activated protein C levels, were measured in an observational study that enrolled patients with AMI-CS, and uncomplicated AMI as controls. The activated protein C is a vitamin K-dependent serine protease that, upon conversion from its inactive form, exerts anticoagulant, anti-inflammatory, and anti-apoptotic effects. Pro-inflammatory cytokines downregulate both the conversion pathway and the endothelial cell protein C receptor, impairing activated protein C effects [28]. The authors reported higher serum levels of IL-6 and CRP in the AMI-CS group that was inversely related to activated protein C levels, which, in contrast, was decreased. While activated protein C was downregulated in all the AMI-CS patients at baseline, the decrease in activated protein C levels was more pronounced in the 28-day non-survivors both on the second and third day after AMI-CS onset. Given the multiple sampling (every day, from day 0, to day 7 after the onset of AMI-CS), a longitudinal correlation between IL-6 and activated protein C was explored. Overall, a strong negative correlation was observed, with the strongest (r^2^= -0.65, *p* < 0.01) being between activated protein C measured on the third day and IL-6 measured on the first day [29]. This study further highlighted the pro-inflammatory role of IL-6, impairing the anti-inflammatory activated protein C pathway.

### IL-6 as a Prognostic Biomarker

IL-6 has been established as an independent predictor of mortality in AMI-CS. Table 1 provides an overview of studies evaluating the prognostic significance of IL-6 in patients with AMI-CS. In a 2006 retrospective study, analysis of stored plasma samples from patients with AMI-CS revealed that a 50 pg/mL increase in IL-6 concentration was associated with a 49% increase in 30-day mortality. Additionally, IL-6 was identified as the sole predictor of 30-day mortality, even after adjusting for potential confounders (including successful revascularization, acute renal failure, serum lactate, SOFA score, and vasopressor dose), with a threshold of 200 pg/mL indicating significantly higher 30-day mortality (with a sensitivity of 74% and specificity of 87%) [10]. IL-6 may also predict organ failure in patients with CS. For instance, another study reported higher levels of IL-6 in CS (63% were AMI-CS) patients complicated by multiorgan failure (MOF) compared to those without MOF. Moreover, CS patients who subsequently developed MOF during the intensive care unit hospitalization exhibited higher IL-6 levels than patients who never developed MOF [30].

**Table 1:** Studies that explored the correlation between IL-6 and outcomes in acute myocardial infarction-related cardiogenic shock (AMI-CS) and the association between IL-6 and the development of cardiogenic shock in patients with AMI

Study, year (Reference)	Design	Population	Sample size	Cytokine studied	Outcomes	Results
Geppert A., 2002 [30]	Retrospective	AMI-CS	51	IL-6	Multiorgan failure	IL-6 levels were higher in patients with CS and multiorgan failure. IL-6 predictor of multiorgan failure
Theroux P., 2005 [40]	Sub-study of randomized trial	STEMI	337	IL-6 and TNF-alpha	90-day mortality	IL-6 is associated with mortality. Both IL-6 and TNF-alpha were associated with death or CS. IL-6 levels were lower after treatment with pexelizumab with lower mortality in the pexelizumab arm
Geppert A., 2006 [10]	Retrospective	AMI-CS	38	IL-6	30-day mortality	IL-6 concentrations above 200 pg/ml are associated with 30-day mortality
Jarai R., 2009 [35]	Retrospective	AMI-CS	58	IL-6	30-day mortality	IL-6 predictor of outcome (patients with both high IL-6 and NT-proBNP had worse outcomes)
Andrié RP., 2012 [34]	Prospective	AMI-CS	87	IL-6	30-day mortality	IL-6 predictor of outcome
Prondzinsky R., 2012 [9]	Sub-study of randomized trial	AMI-CS	40	IL-6 and IL-1beta − 7–8 -10	Mortality or CS	Maximal values of IL-6-8-10 and minimal of IL-7 predict mortality. IL-1beta does not discriminate.
Van Diepen S., 2013 [31]	Sub-study of randomized trial	STEMI	772	IL-6	90-day death, shock, heart failure	IL-6 predictor of clinical outcome
Van Diepen S., 2016 [32]	Sub-study of randomized trial	STEMI	376	IL-6 and IL-10	90-day death, shock, heart failure	IL-10 associated with clinical outcome
Kataja A., 2021 [33]	Retrospective	AMI-CS	183	IL-6	90-day mortality	IL-6 above the median is associated with hypoperfusion and clinical outcome
Ceglarek U., 2021 [36]	Sub-study of randomized trial	AMI-CS	458	IL-6	30-day mortality	IL-6 within CLIP score (cystatin C, NT-proBNP, and lactate) stratified the risk according to the outcome

*Abbreviations: IL*,* interleukin; CS*,* cardiogenic shock; MACE: major adverse cardiovascular events; STEMI: ST segment elevation acute myocardial infarction*

A sub-analysis of the Assessment of Pexelizumab in Acute Myocardial Infarction (APEX-AMI) clinical trial investigated the prognostic relevance of baseline inflammatory biomarkers in patients with STEMI. IL-6 emerged as an independent predictor of clinical outcomes, including AMI-CS. Further, it enhanced the predictive value when incorporated into a clinical prediction model composed of N-terminal pro-brain natriuretic peptide (NT-proBNP), CRP, IL-10, and IL-6 [31]. Subsequently, IL-6 trajectory over 24 h was examined in the same population. While IL-6 level change was inversely associated with successful angiographic reperfusion, it did not show an association with the clinical outcomes, including AMI-CS [32]. Similarly, the CardShock study measured IL-6 levels in 183 patients with CS (AMI-CS was the specific etiology in 79% of the patients), showing that patients with IL-6 levels above the median had more often signs of hypoperfusion and higher 90-day mortality rates [33]. In the same regard, an observational prospective study identified IL-6 as the most robust independent predictor of 30-day mortality, surpassing even age and creatinine [34]. Furthermore, combining IL-6 levels with NT-proBNP yielded enhanced predictive value for 30-day mortality [35]. In 2021, a biomarker-based risk score called “CLIP score”, developed from the CULPRIT-SHOCK trial identified IL-6 alongside cystatin C (a cysteine protease inhibitor used as a biomarker of renal function), lactate, and NT-proBNP as the strongest predictors of 30-day mortality [36]. The intra-aortic balloon pump (IABP) is a mechanical circulatory support device tested in the IABP-SHOCK trial to improve hemodynamics and clinical outcomes in patients with AMI-CS. A subanalysis of this randomized trial found that peak levels of IL-6 were associated with higher mortality in patients with AMI-CS, and its serum levels were not different across the randomization to IABP. Conversely, lower levels of IL-7, which is an immune-regulatory and pro-inflammatory cytokine [20, 22], also predicted mortality. Notably, the use of IABP did not influence any of the inflammatory markers [9]. Despite IL-6 and IL-7 being pro-inflammatory cytokines, their peak levels exhibited inverse trends. This underscores the need for a more comprehensive understanding of the roles of cytokines, where immune-regulatory mechanisms play a crucial part beyond the pro- and anti-inflammatory classification.

The extracorporeal membrane oxygenation is a mechanical circulatory support defined as a life-support technique that provides cardiac and respiratory support by oxygenating the blood, serving as a potential therapeutic option for patients with CS who do not respond to inotropes and other mechanical support strategies. Machine learning clustering was applied to a cohort of patients with CS treated with veno-arterial extracorporeal membrane oxygenation to categorize patients into phenotypes. Three clusters were identified and were defined as follows: “platelet preserved”, “hyperinflammatory” and “hepatic-renal”. The “platelet preserved” cluster was characterized by higher platelet counts, low levels of IL-6 and IL-10, and preserved liver and kidney function. In contrast, the “hyperinflammatory” group, exhibited elevated inflammatory markers and prolonged coagulation times, while the “hepatic-renal,” included patients with severe liver and kidney dysfunction, marked by elevated markers of organ injury and coagulation abnormalities. Interestingly, a random forest classifier determined the five highest predictive variables, with IL-6 being included. IL-6 exhibited the lowest levels in the “platelet preserved” phenotype, while the “hyperinflammatory” phenotype, as was to be expected, showed the highest. Compared to “platelet preserved”, “hyperinflammatory” and “hepatic-renal” phenotypes had higher mortality, with the latter affected by the highest [37].

## IL-6: Mediator or Marker of CS?

IL-6 is primarily expressed by monocytes and endothelial cells [22], which are likely to be activated early in CS. This early activation is supported by findings such as those from Cuinet et al. [12], who observed that CS triggers an acute pro-inflammatory response, followed by tissue repair mechanisms and immune cell depletion. The elevated levels of IL-6 as early as 24 h after CS diagnosis likely suggest a monocytic and endothelial activation at the onset of the condition. The mechanisms underlying the pro-inflammatory effects of IL-6 in CS remain largely unclear. One potential pathway involves the previously discussed downregulation of activated protein C, contributing to the pro-inflammatory environment in CS. These findings highlight IL-6 as a biomarker of systemic inflammation in CS and a potential mediator of its downward spiral pathophysiology. However, the mechanistic link between acute inflammation, hemodynamic impairment, and systemic deterioration is largely unknown.

## Open Questions and Future Directions

To date, no trials have specifically assessed the efficacy of drugs targeting interleukin pathways in the CS setting. However, few trials have specifically assessed the efficacy of drugs targeting the IL-6 pathway in the acute setting of cardiovascular disease, providing early signals supporting its feasibility, thus offering a rationale for further investigation in CS. Specifically, in the Anti-IL-6 treatment in Myocardial Infarction (ASSAIL-MI) trial, a single infusion of tocilizumab was shown to increase myocardial salvage (as measured by magnetic resonance imaging) in patients with ST-segment elevation myocardial infarction compared to placebo [38]. Additionally, a single infusion of tocilizumab was tested in the out-of-hospital cardiac arrest setting, aiming to mitigate systemic inflammation and organ injury in the IMICA Trial. The tocilizumab-treated arm exhibited a significant reduction of C-reactive protein, leukocyte, Troponin T, and NT-proBNP levels [39].

Future research should focus on phenotyping CS patients based on their inflammatory profiles. Identifying specific cytokines linked to distinct clinical scenarios could provide critical insights and enable personalized therapeutic strategies tailored to the underlying inflammatory processes. A key area for exploration is determining whether modulating inflammatory pathways can reduce the inflammatory burden and improve clinical outcomes in CS. Additionally, future studies must address important questions regarding the optimal timing, dosing, and patient populations that would benefit most from IL-6-targeted therapies. Currently, a randomized, double-blind study is underway to evaluate the effects of low-dose dobutamine combined with tocilizumab in AMI patients at high risk for developing CS. (NCT05350592). Lastly, another study is testing the Oxiris membrane™, which removes pro-inflammatory cytokines and lipopolysaccharides (NCT05642273, NCT04886180), in severe CS patients supported with veno-arterial extracorporeal membrane oxygenation.

## Conclusions

CS represents a life-threatening condition and systemic inflammation is a major contributor to both its progression and adverse outcomes. Elevated IL-6 levels are associated with the extent of organ dysfunction and high mortality rates, highlighting its potential as both a risk biomarker and therapeutic target in CS. Mitigating inflammation may represent a promising path in CS. However, further studies are needed to determine the optimal timing, modality, and patient populations for the practical application of anti-inflammatory therapies. Clinical trials of IL-6 blockade in cardiogenic shock are lacking.

## Data Availability

No datasets were generated or analysed during the current study.

## Key References

- Samsky MD, Morrow DA, Proudfoot AG, Hochman JS, Thiele H, Rao SV. Cardiogenic Shock After Acute Myocardial Infarction: A Review. Jama. 2021;326(18):1840-50
  **This review highlights the impact of AMI-CS being the leading cause of post-AMI mortality.**
- Cuinet J, Garbagnati A, Rusca M, Yerly P, Schneider AG, Kirsch M, et al. Cardiogenic shock elicits acute inflammation, delayed eosinophilia, and depletion of immune cells in most severe cases. Sci Rep. 2020;10(1):7639
  **Findings from this study demonstrated how CS elicits an acute pro-inflammatory response, analyzing longitudinal mediator changes, including IL-6.**
- Ceglarek U, Schellong P, Rosolowski M, Scholz M, Willenberg A, Kratzsch J, et al. The novel cystatin C, lactate, interleukin-6, and N-terminal pro-B-type natriuretic peptide (CLIP)-based mortality risk score in cardiogenic shock after acute myocardial infarction. Eur Heart J. 2021;42(24):2344-52.
  **This manuscript incorporated IL-6 levels into a novel mortality risk score in patients with AMI-CS.**

## References

[1] Diepen Sv, Katz JN, Albert NM, Henry TD, Jacobs AK, Kapur NK, et al. Contemporary management of cardiogenic shock: A scientific statement from the American heart association. Circulation. 2017;136(16):e232–68. 28923988 10.1161/CIR.0000000000000525

[2] Samsky MD, Morrow DA, Proudfoot AG, Hochman JS, Thiele H, Rao SV. Cardiogenic shock after acute myocardial infarction: A review. JAMA. 2021;326(18):1840–50. 34751704 10.1001/jama.2021.18323 PMC9661446

[3] Jentzer JC. Understanding Cardiogenic Shock Severity and Mortality Risk Assessment. Circulation: Heart Failure. 2020;13(9):e007568. 10.1161/CIRCHEARTFAILURE.120.007568 32900232

[4] Møller JE, Engstrøm T, Jensen LO, Eiskjær H, Mangner N, Polzin A, et al. Microaxial flow pump or standard care in Infarct-Related cardiogenic shock. N Engl J Med. 2024;390(15):1382–93. 38587239 10.1056/NEJMoa2312572

[5] Thiele H, Zeymer U, Akin I, Behnes M, Rassaf T, Mahabadi AA, et al. Extracorporeal life support in Infarct-Related cardiogenic shock. N Engl J Med. 2023;389(14):1286–97. 37634145 10.1056/NEJMoa2307227

[6] Sinha SS, Morrow DA, Kapur NK, Kataria R, Roswell RO. 2025 Concise Clinical Guidance: An ACC Expert Consensus Statement on the Evaluation and Management of Cardiogenic Shock. JACC. 2025;85(16):1618-41. 10.1016/j.jacc.2025.02.018 40100174

[7] Hochman JS. Cardiogenic shock complicating acute myocardial infarction. Circulation. 2003;107(24):2998–3002. 12821585 10.1161/01.CIR.0000075927.67673.F2

[8] Silvis MJM, Kaffka Genaamd Dengler SE, Odille CA, Mishra M, van der Kaaij NP, Doevendans PA, et al. Damage-Associated molecular patterns in myocardial infarction and heart transplantation: the road to translational success. Front Immunol. 2020;11:599511. 33363540 10.3389/fimmu.2020.599511 PMC7752942

[9] Prondzinsky R, Unverzagt S, Lemm H, Wegener NA, Schlitt A, Heinroth KM, et al. Interleukin-6, -7, -8 and– 10 predict outcome in acute myocardial infarction complicated by cardiogenic shock. Clin Res Cardiol. 2012;101(5):375–84. 22212516 10.1007/s00392-011-0403-3

[10] Geppert A, Dorninger A, Delle-Karth G, Zorn G, Heinz G, Huber K. Plasma concentrations of interleukin-6, organ failure, vasopressor support, and successful coronary revascularization in predicting 30-day mortality of patients with cardiogenic shock complicating acute myocardial infarction. Crit Care Med. 2006;34(8):2035–42. 16775569 10.1097/01.CCM.0000228919.33620.D9

[11] Debrunner M, Schuiki E, Minder E, Straumann E, Naegeli B, Mury R, et al. Proinflammatory cytokines in acute myocardial infarction with and without cardiogenic shock. Clin Res Cardiol. 2008;97(5):298–305. 18491171 10.1007/s00392-007-0626-5

[12] Cuinet J, Garbagnati A, Rusca M, Yerly P, Schneider AG, Kirsch M, et al. Cardiogenic shock elicits acute inflammation, delayed eosinophilia, and depletion of immune cells in most severe cases. Sci Rep. 2020;10(1):7639. 32377009 10.1038/s41598-020-64702-0 PMC7203157

[13] Kohsaka S, Menon V, Lowe AM, Lange M, Dzavik V, Sleeper LA, et al. Systemic inflammatory response syndrome after acute myocardial infarction complicated by cardiogenic shock. Arch Intern Med. 2005;165(14):1643–50. 16043684 10.1001/archinte.165.14.1643

[14] Pudil R, Krejsek J, Pidrman V, Gregor J, Tichý M, Bures J. Inflammatory response to acute myocardial infarction complicated by cardiogenic shock. Acta Medica (Hradec Kralove). 2001;44(4):149–51. 11836852

[15] Jentzer JC, Bhat AG, Patlolla SH, Sinha SS, Miller PE, Lawler PR, et al. Concomitant sepsis diagnoses in acute myocardial Infarction-Cardiogenic shock: 15-Year National Temporal trends, management, and outcomes. Crit Care Explor. 2022;4(2):e0637. 35141527 10.1097/CCE.0000000000000637 PMC8820909

[16] van Diepen S, Vavalle JP, Newby LK, Clare R, Pieper KS, Ezekowitz JA, et al. The systemic inflammatory response syndrome in patients with ST-segment elevation myocardial infarction. Crit Care Med. 2013;41(9):2080–7. 23760155 10.1097/CCM.0b013e31828a67b2

[17] Jentzer JC, Lawler PR, Diepen Sv, Henry TD, Menon V, Baran DA et al. Systemic Inflammatory Response Syndrome Is Associated With Increased Mortality Across the Spectrum of Shock Severity in Cardiac Intensive Care Patients. Circulation: Cardiovascular Quality and Outcomes. 2020;13(12):e006956. 10.1161/CIRCOUTCOMES.120.006956 33280435

[18] Lim N, Dubois MJ, De Backer D, Vincent JL. Do all nonsurvivors of cardiogenic shock die with a low cardiac index? Chest. 2003;124(5):1885–91. 14605064 10.1378/chest.124.5.1885

[19] Dinarello CA. Immunological and inflammatory functions of the interleukin-1 family. Annu Rev Immunol. 2009;27:519–50. 19302047 10.1146/annurev.immunol.021908.132612

[20] Dinarello CA. Historical insights into cytokines. Eur J Immunol. 2007;37(Suppl 1):S34–45. 17972343 10.1002/eji.200737772 PMC3140102

[21] Abbate A, Toldo S, Marchetti C, Kron J, Van Tassell BW, Dinarello CA. Interleukin-1 and the inflammasome as therapeutic targets in cardiovascular disease. Circ Res. 2020;126(9):1260–80. 32324502 10.1161/CIRCRESAHA.120.315937 PMC8760628

[22] Akdis M, Aab A, Altunbulakli C, Azkur K, Costa RA, Crameri R, et al. Interleukins (from IL-1 to IL-38), interferons, transforming growth factor β, and TNF-α: receptors, functions, and roles in diseases. J Allergy Clin Immunol. 2016;138(4):984–1010. 27577879 10.1016/j.jaci.2016.06.033

[23] Heinrich PC, Castell JV, Andus T. Interleukin-6 and the acute phase response. Biochem J. 1990;265(3):621–36. 1689567 10.1042/bj2650621 PMC1133681

[24] Garlanda C, Dinarello Charles A, Mantovani A. The Interleukin-1 family: back to the future. Immunity. 2013;39(6):1003–18. 24332029 10.1016/j.immuni.2013.11.010 PMC3933951

[25] Black S, Kushner I, Samols D. C-reactive protein**. J Biol Chem. 2004;279(47):48487–90. 15337754 10.1074/jbc.R400025200

[26] Rose-John S. IL-6 trans-signaling via the soluble IL-6 receptor: importance for the pro-inflammatory activities of IL-6. Int J Biol Sci. 2012;8(9):1237–47. 23136552 10.7150/ijbs.4989 PMC3491447

[27] Selejan S, Pöss J, Walter F, Hohl M, Kaiser R, Kazakov A, et al. Ischaemia-induced up-regulation of Toll-like receptor 2 in Circulating monocytes in cardiogenic shock. Eur Heart J. 2012;33(9):1085–94. 21998404 10.1093/eurheartj/ehr377

[28] Griffin JH, Fernández JA, Gale AJ, Mosnier LO. Activated protein C. J Thromb Haemost. 2007;5(Suppl 1):73–80. 17635713 10.1111/j.1538-7836.2007.02491.x

[29] Fellner B, Rohla M, Jarai R, Smetana P, Freynhofer MK, Egger F, et al. Activated protein C levels and outcome in patients with cardiogenic shock complicating acute myocardial infarction. Eur Heart J Acute Cardiovasc Care. 2017;6(4):348–58. 26936869 10.1177/2048872616637036

[30] Geppert A, Steiner A, Zorn G, Delle-Karth G, Koreny M, Haumer M, et al. Multiple organ failure in patients with cardiogenic shock is associated with high plasma levels of interleukin-6. Crit Care Med. 2002;30(9):1987–94. 12352031 10.1097/00003246-200209000-00007

[31] van Diepen S, Newby LK, Lopes RD, Stebbins A, Hasselblad V, James S, et al. Prognostic relevance of baseline pro- and anti-inflammatory markers in STEMI: an APEX AMI substudy. Int J Cardiol. 2013;168(3):2127–33. 23394896 10.1016/j.ijcard.2013.01.004

[32] van Diepen S, Alemayehu WG, Zheng Y, Theroux P, Newby LK, Mahaffey KW, et al. Temporal changes in biomarkers and their relationships to reperfusion and to clinical outcomes among patients with ST segment elevation myocardial infarction. J Thromb Thrombolysis. 2016;42(3):376–85. 27324144 10.1007/s11239-016-1390-z

[33] Kataja A, Tarvasmäki T, Lassus J, Sionis A, Mebazaa A, Pulkki K, et al. Kinetics of procalcitonin, C-reactive protein and interleukin-6 in cardiogenic shock - Insights from the cardshock study. Int J Cardiol. 2021;322:191–6. 32841617 10.1016/j.ijcard.2020.08.069

[34] Andrié RP, Becher UM, Frommold R, Tiyerili V, Schrickel JW, Nickenig G, et al. Interleukin-6 is the strongest predictor of 30-day mortality in patients with cardiogenic shock due to myocardial infarction. Crit Care. 2012;16(4):R152. 22889197 10.1186/cc11467 PMC3580741

[35] Jarai R, Fellner B, Haoula D, Jordanova N, Heinz G, Karth GD, et al. Early assessment of outcome in cardiogenic shock: relevance of plasma N-terminal pro-B-type natriuretic peptide and interleukin-6 levels. Crit Care Med. 2009;37(6):1837–44. 19384228 10.1097/CCM.0b013e31819fe896

[36] Ceglarek U, Schellong P, Rosolowski M, Scholz M, Willenberg A, Kratzsch J, et al. The novel Cystatin C, lactate, interleukin-6, and N-terminal pro-B-type natriuretic peptide (CLIP)-based mortality risk score in cardiogenic shock after acute myocardial infarction. Eur Heart J. 2021;42(24):2344–52. 33647946 10.1093/eurheartj/ehab110

[37] Wang S, Wang L, Du Z, Yang F, Hao X, Wang X, et al. Phenotyping refractory cardiogenic shock patients receiving Venous-Arterial extracorporeal membrane oxygenation using machine learning algorithms. Rev Cardiovasc Med. 2024;25(8):303. 39228471 10.31083/j.rcm2508303 PMC11367001

[38] Broch K, Anstensrud AK, Woxholt S, Sharma K, Tøllefsen IM, Bendz B, et al. Randomized trial of Interleukin-6 receptor Inhibition in patients with acute ST-Segment elevation myocardial infarction. J Am Coll Cardiol. 2021;77(15):1845–55. 33858620 10.1016/j.jacc.2021.02.049

[39] Meyer MAS, Wiberg S, Grand J, Meyer ASP, Obling LER, Frydland M, et al. Treatment effects of Interleukin-6 receptor antibodies for modulating the systemic inflammatory response after Out-of-Hospital cardiac arrest (The IMICA Trial): A Double-Blinded, Placebo-Controlled, Single-Center, Randomized, Clinical Trial. Circulation. 2021;143(19):1841–51. 33745292 10.1161/CIRCULATIONAHA.120.053318 PMC8104015

[40] Théroux P, Armstrong PW, Mahaffey KW, Hochman JS, Malloy KJ, Rollins S, et al. Prognostic significance of blood markers of inflammation in patients with ST-segment elevation myocardial infarction undergoing primary angioplasty and effects of pexelizumab, a C5 inhibitor: a substudy of the COMMA trial. Eur Heart J. 2005;26(19):1964–70. 15872036 10.1093/eurheartj/ehi292

